# Anatomy of Maxillary Sinus: Focus on Vascularization and Underwood Septa via 3D Imaging

**DOI:** 10.3390/tomography10040034

**Published:** 2024-03-24

**Authors:** Sara Bernardi, Serena Bianchi, Davide Gerardi, Pierpaolo Petrelli, Fabiola Rinaldi, Maurizio Piattelli, Guido Macchiarelli, Giuseppe Varvara

**Affiliations:** 1 Department of Life, Health and Environmental Sciences, University of L’Aquila, 67100 L’Aquila, Italy; serena.bianchi@univaq.it (S.B.); davide.gerardi@graduate.univaq.it (D.G.); guido.macchiarelli@univaq.it (G.M.); 2 Department of Innovative Technologies in Medicine & Dentistry, Dental School, ‘G. D’Annunzio’ University of Chieti-Pescara, 66100 Chieti, Italy; pierpipetre92@hotmail.it (P.P.); fabiola.rinaldi@studenti.unich.it (F.R.); mpiattel@unich.it (M.P.); gvarvara@unich.it (G.V.)

**Keywords:** maxillary sinus, CBCT, alveolar antral artery, Underwood’s septa

## Abstract

The study of the maxillary sinus anatomy should consider the presence of two features of clinical importance. The arterial supply course and the presence of the so-called Underwood septa are two important factors to consider when planning surgical treatment to reduce the risk of surgical complications such as excessive bleeding and Schneiderian membrane perforations. This study aimed to investigate the above-mentioned anatomical structures to improve the management of eventual vascular and surgical complications in this area. This study included a total of 200 cone-beam computed topographies (CBCTs) divided into two groups of 100 CBCTs to evaluate the arterial supply (AAa) course through the lateral sinus wall and Underwood’s septa, respectively. The main parameters considered on 3D imaging were the presence of the AAa in the antral wall, the length of the arterial pathway, the height of the maxillary bone crest, the branch sizes of the artery in the first group, and the position of the septa, the length of the septa, and their gender associations in the second group. The CBCT analysis showed the presence of the arterial supply through the bone wall in 100% of the examined patients, with an average size of 1.07 mm. With regard to the septa, 19% of patients presented variations, and no gender difference was found to be statistically significant. The findings add to the current understanding of the clinical structure of the maxillary sinus, equipping medical professionals with vital details for surgical preparation and prevention of possible complications.

## 1. Introduction

The head region is one of the richest and most interesting regions in the human body, with nervous, sensorial, respiratory, and digestive organs and the maxillary sinuses found in the maxillary bone, which has a shape and a position that can put them in close proximity to the oral cavity, nasal cavity, and orbital cavity. Historically, the maxillary sinus was identified in Ancient Egypt, together with its relationship with the teeth, and later on, it was described in depth by Leonardo Da Vinci, Nathaniel Highmore, and Schneider [1,2,3].

Anatomical knowledge of the maxillary sinus has clinical relevance for the understanding of the pathogenesis of inflammatory and oncologic diseases involving it and the anatomical structures near it [4,5,6]: pre-clinical evaluations of maxillary sinus anatomy and conditions are mandatory for diagnosis and surgical planning, in cases of functional endoscopy surgeries, floor lift, volume augmentation, and dental implant placement.

**Figure 1:**
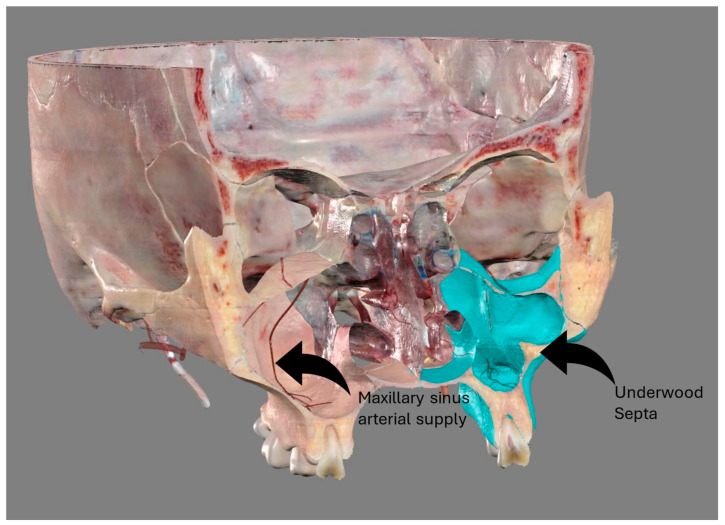
Anatomical features of the maxillary sinus. Image obtained from Anatomage^®^, high-resolution regions. On the right, through the sinus it is possible to appreciate the arterial supply of the maxillary sinus walls. On the left, the presence of a septum.

The introduction of cone beam computed tomography (CBCT) into clinical practice allows for the surgical planning of procedures such as sinus floor augmentation and implant placement in the maxillary bone: the crucial features of the maxillary sinus (Figure 1) studied using CBCT are the position of the anastomosis of the posterior alveolar artery supplying the lateral wall of the sinus (hereafter referred to as AAa), the presence of Underwood’s septa, and the opening of the nasal ostium [7,8].

Indeed, preserving the AAa is important for guaranteeing the right vascular supply for eventual grafting procedures and integrations, as well as for avoiding excessive bleeding during the surgical procedure [9,10,11,12,13,14].

The vascular supply of paranasal sinuses is also crucial in functional endoscopy sinus surgery (FESS), which is a predictable procedure to treat odontogenic and non-odontogenic sinusitis [5,6]. With regard to the FESS procedure, the course and derivation of the AAa should be considered to guarantee visibility during the procedure.

Underwood’s septa are dense bone projections in the maxillary sinus, which can complicate surgery in several ways [2,3,7,8,15,16]: the maxillary septa can prevent adequate access and visualization of the sinus floor, and the graft may result in being insufficient or incomplete. During a sinus floor lift with the lateral access approach, the window may not fracture, but it can rotate in its medial position. The presence of bone facilitates the tearing of the membrane during the release of the access window [2,3,7,8,15,16]. In addition, during the procedure for retriving foreing bodies, including Caldwell–Luc interventions or FESS, Underwood’s septa can limit the maneuvers and the visibility of the operator [5,6].

However, the cortical bone nature of the septa can allow for an immediate implant placement, and the septa may be responsible for faster bone formation, as they act as an additional wall of bone for the proliferation of blood vessels within the graft.

The aim of this study is to investigate the arterial blood supply of the maxillary sinus and the presence of Underwood’s septa, using CBCTs and providing high-quality CBCT renderings. The information outlined in this study aims to provide clinicians with an updated and a particular visualization review of maxillary sinus clinical anatomy of which in-depth knowledge is important to avoid complications which can result in diseases that have therapies requiring further and invasive surgical intervention.

## 2. Materials and Methods

### 2.1. Ethics

The results of this study are part of a research project on anatomical variants in the oral anatomy. This study was conducted in compliance with the Declaration of Helsinki and approved by the Inter-Institutional Ethics Committee of the G. D’Annunzio University of Chieti-Pescara (protocol n. 27. Approved in December 2021).

### 2.2. Sample and Study Design

This study included all patients referred to the Dental Clinic of the University “G. d’Annunzio” Chieti-Pescara. Inclusion criteria included patients who, after the usual medical history and dental situation assessment, needed a second-level radiological exam to confirm a diagnosis and to establish a treatment plan. The exclusion criteria were the patient’s opposition to involvement in this study and patient < 18 years of age.

The selection of CBCT images was carried out through a random sampling of the radiological database for diagnosis and surgical treatment, collecting 100 radiograms from the selected population based on the inclusion and exclusion criteria described above to evaluate the incidence, frequency, and characteristics of the alveolar antral artery and anatomy of and variations in the number of Underwood’s septa in the maxillary sinus, respectively. Images were obtained using the CBCT scanner PaX-Zenith3D (Vatech, 13, Samsung 1-ro 2-gil, Hwaseong-si, Gyeonggi-do, 445-170, Korea) with a voxel size of 0.3 mm.

CBCT measurements were carried out by two authors [D.G. (Doctor in Dental Surgery) and S.B. (Doctor in Dental Surgery, PhD in Imaging, Assistant Professor in Anatomy)]. DICOM files were imported and studied in 3 dimensions using Ez3D plus Premium software ver. 1.2.6.2 (Vatech, 13, Samsung 1-ro 2-gil, Hwaseong-si, Gyeonggi-do, 445-170, Korea) on axial, sagittal, transverse, and panoramic views for all hemi-mandibles, following standardized viewing settings, with field of view ranging from 5 × 5 cm to 24 × 19 cm.

This study was conducted according to the Anatomical Quality Assurance (AQUA) checklist [17].

### 2.3. Evaluation of AAa

A total of 100 CBCTs were investigated, performed between 2012 and 2019, belonging to 100 patients who had a clinical condition of edentulism from first premolars to first molars, thus two maxillary sinuses, with a height of the bone crest up to 5 mm, eligible for surgical treatment of maxillary sinus augmentation through lateral approach. The observations and measurements were carried out using CBCT evaluating the same parameters taken from the study of Bernardi et al. [18].

**Figure 2:**
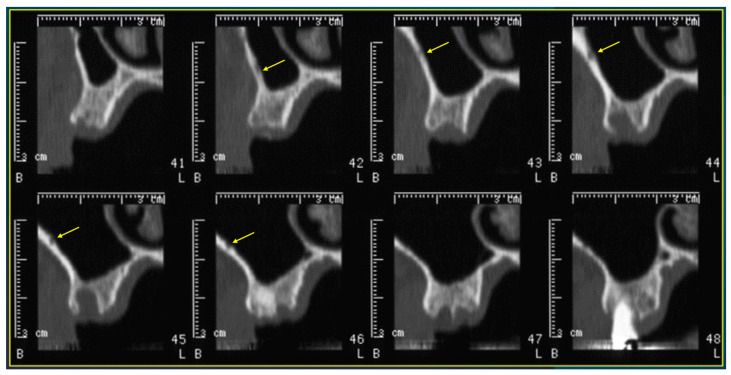
Cross-sections showing the AAa pathway, indicated by the yellow arrow.

**Figure 3:**
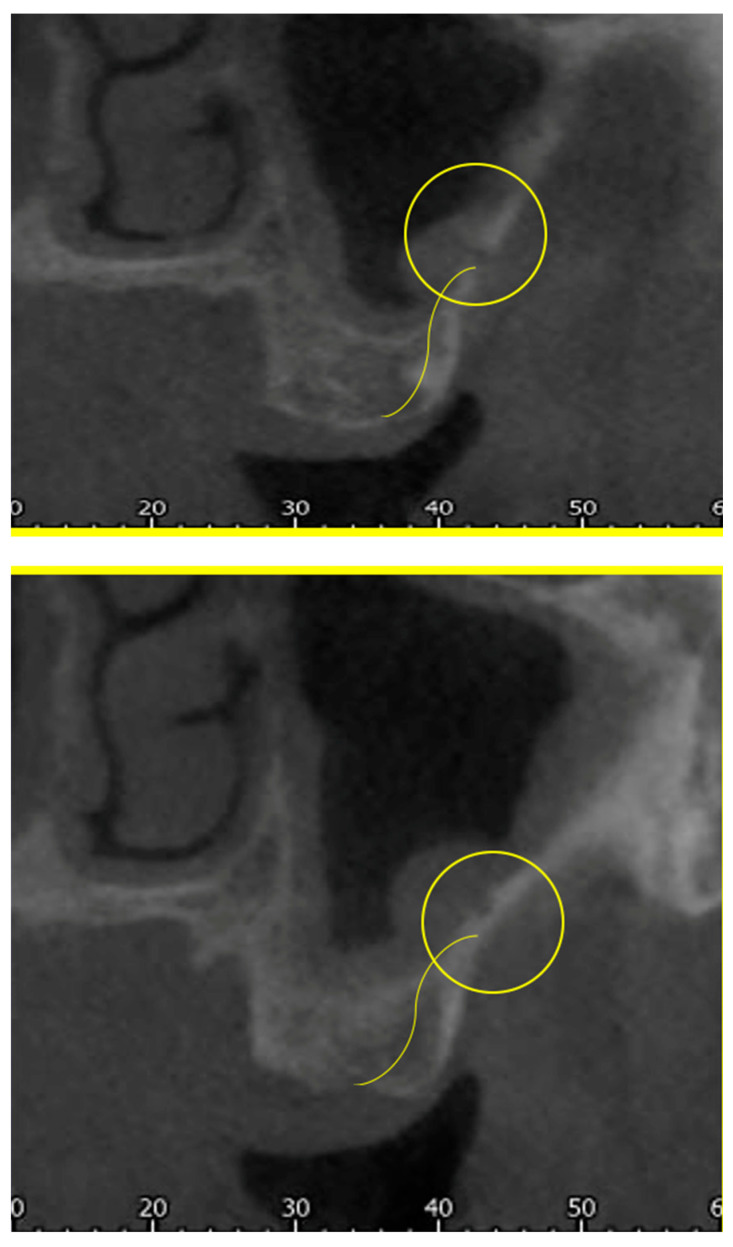
Measure of the cranio-caudal distance of the AAa from the bone crest.

In particular, the following parameters were evaluated:

1. The presence of the AAa in the antral wall of the maxillary sinus;
2. The length of the arterial pathway in the mesiodistal dimension, on cross-section setting at 1 mm (Figure 2);
3. The height of the maxillary bone crest in the cranio-caudal direction, on cross-section at the level of the first superior molar (Figure 3);
4. The sized branches of the alveolar antral artery at the level of the first superior molar.

### 2.4. Statistics

Statistical analyses were performed using Chi-square to assess left-right and gender differences. The Student’s *t*-test was used to compare left and right lengths and gauges. The alpha was set at 0.05.

The null hypothesis included no differences between genders and hemiarches and was considered rejected if the *p*-value was ≤0.05. The tests were performed using the SAS Studio University Edition software, USA.

### 2.5. Evaluation of Underwood Septa

A sample of 100 CBCTs, different from the sample used for evaluation of AAa, performed between 2012 and 2020, was collected, with a total of 200 maxillary sinuses. The observations and measurements were carried out using the same parameters as the study of Sakhdari et al. [19].

**Figure 4:**
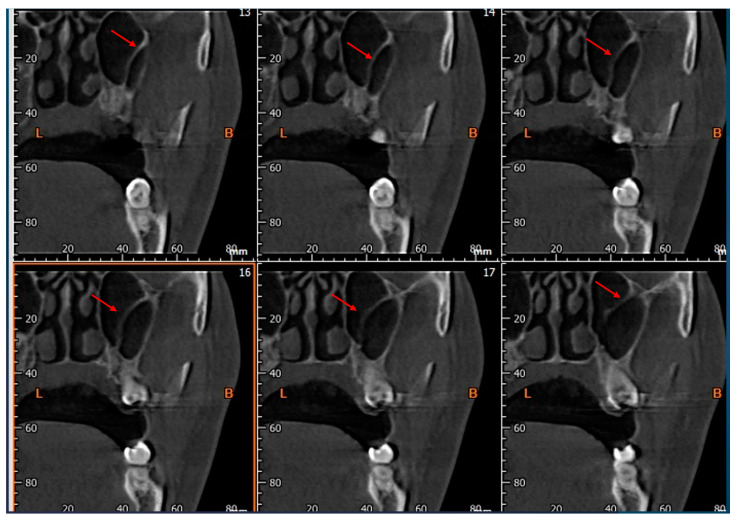
Cross-sections showing the presence of Underwood’s septa, indicated by the red arrow.

The following parameters concerning Underwood’s septa were evaluated:

1. The presence of the septa on cross-section (Figure 4);
2. The height of the septa on cross-section setting from 2 mm to 0 mm, to better evaluate the maxillary sinus: this was measured from the apex of the septa to its bottom on the basal bone;
3. The localization of the septa on cross-section;
4. The orientation of the septa.

### 2.6. Statistics

The variables statistically analyzed were: (1) the position of the septa detected according to Underwood’s classification and the orientation of the septa; (2) the mean and the standard deviation of the length of the septa; (3) any gender associations, using Fisher’s exact test. The alpha was set at 0.05. The null hypothesis included that there were no differences between genders and considered rejected if the *p*-value was ≤0.05. The tests were performed using the SAS Studio University Edition software, USA.

## 3. Results

**Figure 5:**
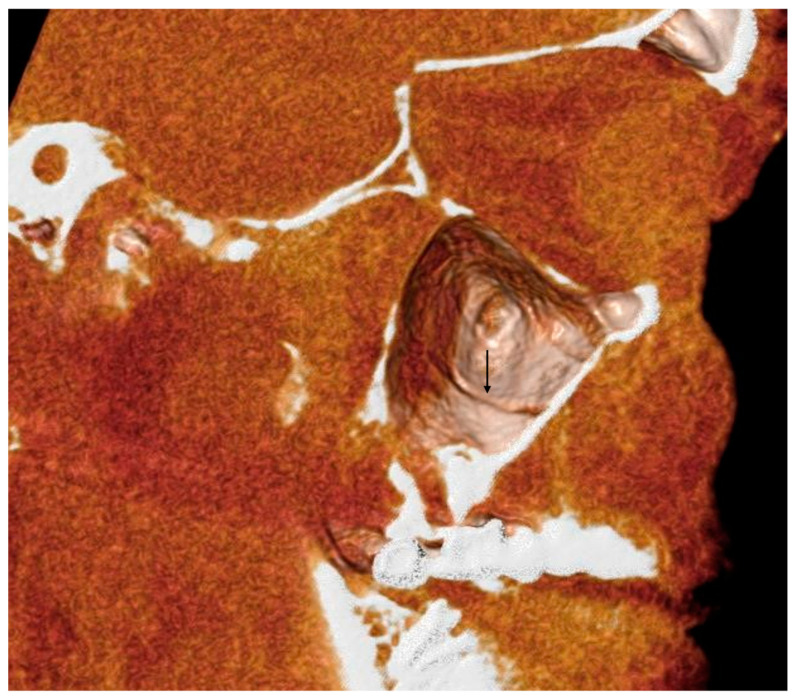
A 3D volume rendering of an analyzed CBCT. Image obtained from Anatomage^®^. It is possible to appreciate the groove of the AAa, indicated by the black arrow.

**Figure 6:**
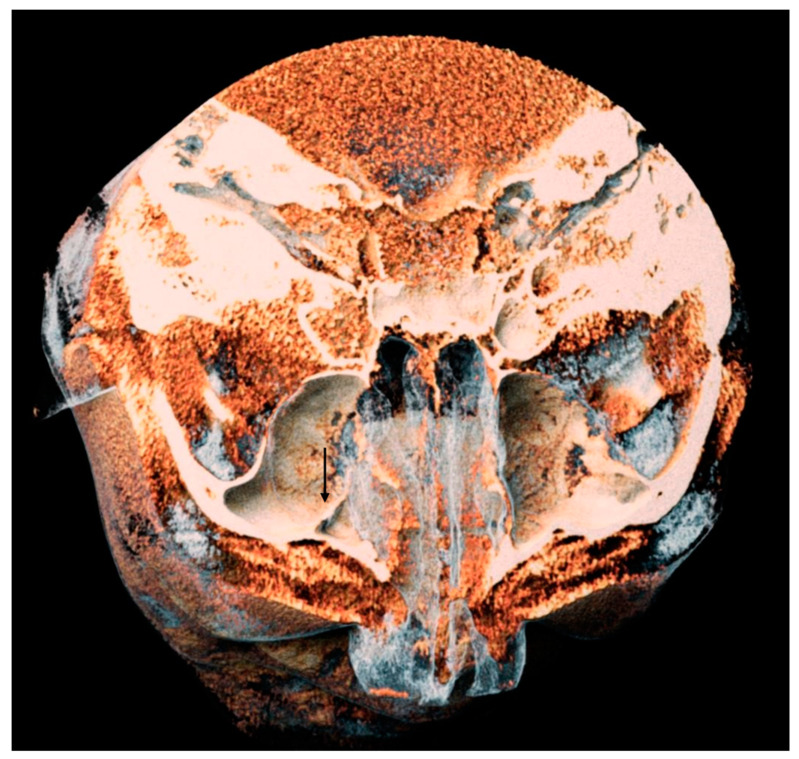
A 3D volume rendering of an analyzed CBCT. Image obtained from Anatomage^®^. It is possible to appreciate on the left side a septum of the maxillary sinus, indicated by the black arrow.

This study’s results highlighted the peculiar characteristics of the maxillary sinus’s vascular supply and its morphological variants (Figure 5 and Figure 6).

### 3.1. AAa Presence and Morphometry

The sample of patients was represented by 42% male patients and 58% female patients. The mean age was 68.46 ± 9.66 years old (43–88 years old). The chi-square test showed no statistically significant differences between the presence on the right and left hemiarch (Table 1).

**Figure 7:**
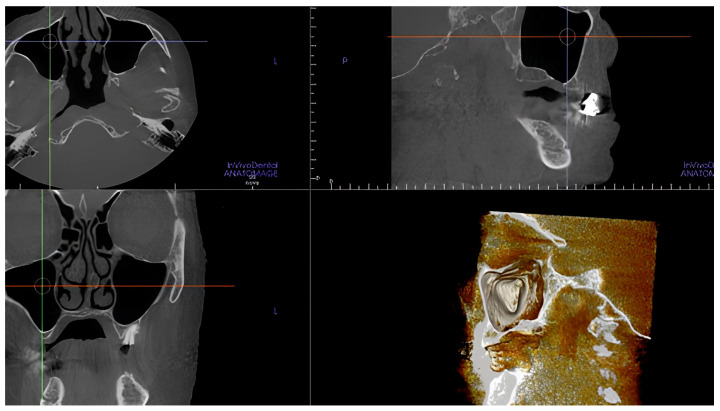
CBCT scans and volume rendering using in vivo dental Anatomage showing the artery was not detectable.

The presence of the intraosseous AAa was 100% in the sample examined. Based on the differences between the right and left hemiarches, we can state that the posterior alveolar artery that supplies them is radiologically detectable in 99% of the cases (Figure 7).

The considered extension as the distal mesiodistal course presents an average value on the right of 24.06 ± 5.66 mm while on the left it presents an average value of 23.21 ± 5.43 mm. The cranio-caudal distance from the crest on the right hemiarch is 12.25 ± 3.84 mm, while on the left, it is 13.44 ± 3.91 mm (Table 2). Of 200 cases, 40 (20%) had a distance greater than 15 mm in the first molar region. The average caliber is around 1.07 mm and rarely reaches 2 mm (Table 1).

The minimum caliber recorded was 0.4 mm, and the maximum was 1.8 mm, with recorded values almost always between 0 and 2 mm, rarely exceeding 2 mm (Table 2), specifically:

- 48% with a gauge of less than 1 mm;
- 51% with a gauge between 1 and 2 mm;
- 1% with a gauge greater than 2 mm.

### 3.2. Differences between Left and Right Hemiarches and between Genders

Regarding the differences between the right and left hemiarch, the results of the Student’s *t*-test showed that the length of the left mesiodistal intraosseous course is significantly greater than the one on the right side (>1 mm) (Table 1).

The cranio-caudal distance of the right AAa in women averaged 12.67 mm, while in men it was 11.66 mm. In addition, it was found that on the left, the cranio-caudal distance of the AAa in women averaged 13.46 mm, while in men, it was 13.43 mm (Table 3), with no statistically significant difference.

The evaluation of the mesiodistal extension of the two hemiarches with the two genders considered showed that in women, on the right hemiarch, the artery extends 23.94 mm while in men it extends 24.22 mm (Table 3). Considering the extension of the left hemiarch, in women the arterial vessel was found to be on average 22.49 mm while in men it was found to be 24.14 mm (Table 3), with no statistically significant difference.

Considering the variable of the caliber (Table 4) of the two hemiarches and considering the gender, the right side of the female gender showed an average size of 1 mm. In contrast, in the male gender, it was 1.18 mm. Regarding the left side, the artery in the female gender offered a mean caliber of 1.02 mm, whereas in the male gender, it was 1.13 mm. The caliber in the right hemiarch is statistically significant, being greater in male subjects (*p*-value < 0.05).

### 3.3. Underwood’s Septa Presence

The sample included 50% male patients and 50% female patients. The mean age was 57.73 years old (range 40–80 years old).

The presence of septa within the maxillary sinus was 19% in the examined sample (Table 5).

On the right side, they are present in 7% and in the left side in 4% in those with single septa; meanwhile, in those with double septa, it is observed that in all the patients (4%) the septa are all located on the right side, and in 3% female and 1% male (Table 6).

### 3.4. Underwood’s Septa Position and Morphometry

According to Underwood’s classification, the position of the septum can be anterior, central, or posterior. It is observed that single septa in the right sinus are predominantly located anteriorly. It is also observed that the septa in the anterior and posterior positions are found in the male sex. At the same time, those in the middle position are predominantly located in the female sex. Image analysis on the left side showed the single septa are predominantly located in the anterior position, while the septa in the posterior position are absent (Table 7).

The double septa are predominantly located in the female sex and in the central position.

We can also see the absence of double septa in both genders on the left side (Table 8).

The septa were measured from their apex to the basal bone. The septa detected in the male right maxillary sinuses measured on average 17.18 ± 7.76 mm; the single septa detected in the female right maxillary sinuses measured 6.5 ± 0.49 mm.

The left single septa detected in female patients measured an average of 11 ± 2.8 mm. The double septa measured in the female right maxillary sinuses measured 13.16 ± 6.9 mm and 7.4 ± 1.8 mm, respectively.

## 4. Discussion

### 4.1. The Maxillary Sinus Arterial Vascularization

Several researchers have studied the blood supply of the maxillary sinus for the last fifteen years. Using cadaver and radiological models, they have assessed and described the arterial course within the lateral wall of the maxillary bone and the Schneider membrane.

Traxler et al. and Solar et al. 1999 conducted a cadaver study evaluating the maxillary sinus vascularization in edentulous specimens [9,10]: they found the posterior superior alveolar artery and the infraorbital artery, using an intraosseous and extraosseous anastomosis, supplying the lateral wall of the maxillary sinus and the alveolar process: this anastomosis has been described as providing extra-osseous branches with a mean caliber of 1.6 mm and a length of 44.6 mm [9,10]. In 2011, Rosano et al. conducted a cadaver and a living study using CT: the cadaver study showed the presence of anastomosis in 100% of the examined sample, while the frequency in the radiological study was 47%. In most cases (55%), the mean caliber was less than 1 mm [20]. Jung et al., in 2011, similarly conducted a study on CBCT, finding a frequency of 52.5% of the anastomosis, mainly in the sub-membranous position. In 2013, several studies were published on this topic: Anamali et al. [21], Apostolakis et al. [22], and Ilguy et al. [23] reported the results of investigations on this arterial anastomosis using CBCT and reporting a frequency of 97.2%, 82%, and 52.8%, respectively. Hayek et al. [24] in 2015 found a frequency of 50%, and in 2016, Bernardi et al. [18], Lee et al. [25], Danesh-Sani et al. [26], Pandharbale et al. [27], and Verela Centelles et al. [28] found a frequency of 35%, 32%, 60.58%, 72%, 93%, and 52%, respectively. As regards the diameters, Bernardi et al. [18], Danesh-Sani et al. [26], Pandharbale et al. [27], and Verela Centelles et al. [28] found a mean diameter of 1.61, 1.17, 0.63, and 1.35 mm, respectively. In 2017, Al Ghurabi et al. [29], Chitsazi et al. [30], Lozano-Carrascal et al. [31], Panjnoush et al., San Aung et al. [32], and Tehranchi et al. [33], found a frequency of 90%, 35%, 48.6%, 25%, 93%, and 87%, respectively.

As regards the diameters, Chitsazi et al. [30] and Tehranchi et al. [33] found a mean diameter of 1.37 mm and 1.29 mm, respectively. In 2018, Şimşek Kaya et al. [34], Rostetter et al. [35], and Sun et al. [36] found a frequency of 87.7%, 92%, and 87.6%, respectively. Şimşek Kaya et al. [34] reported the mean of the diameters as 1.04 mm. The studies published in 2019 by Amine et al. [8], Yalcin et al. [37], and Yusof et al. [38] found a frequency of 97.3%, 72.2%, and 63.7%, respectively. As regards the mean diameters, Yusof et al. [38] reported that in the dentate patients, the mean caliber was 1.4 mm, and in edentulous patients, 1.0 mm. Albuquerque et al. [39], Alves et al. [40], Dias et al. [41], Padovani et al. [42], and Tran et al. [43] in 2020 found a frequency of 88.5%, 89.3%, 56.2%, 76.7%, and 84%, respectively. As regards the parameter of the mean diameter, Albuquerque et al. found a measure of 1.32 mm, while Alves et al. found a measure of 1.16 mm, and Tran et al. [43] found an average diameter of 0.91 mm. Finally, in 2021, Fayek et al. [44] and Godil et al. [45] found a frequency of 92% with a mean diameter of 1.0 mm and a frequency of 99.4%, respectively.

In the systematic review and meta-analysis of Alves et al. [46], the authors found the frequency of the anastomosis at 74%, using imaging studies with a difference between the CBCT studies and the CT studies: the authors attributed this difference to the FOV selections and the voxel size [46]. Furthermore, the diameters’ data fell in the range indicated in the literature: the authors reported that most of the investigated arteries have a diameter ranging from 1 to 1.9 mm [46].

Our findings revealed that the intraosseous course of AAa was present in all cases examined: the artery was radiologically detectable in almost all cases, with no significant differences between the right and left sides; this information is crucial for surgical procedures, as preserving the AAa is essential for ensuring adequate vascular supply during grafting procedures and avoiding excessive bleeding. We also observed that the length of the AAa in the mesiodistal dimension and the cranio-caudal distance from the crest varied slightly between the right and left sides. However, these differences were not statistically significant. The caliber of the AAa branches in the first superior molar area mainly ranged between 1 and 2 mm, with a few cases exceeding 2 mm: this finding is important for clinicians to be aware of the artery size when planning surgical procedures. Interestingly, we found that the caliber of the AAa was slightly higher in male subjects than in female subjects. This difference may be attributed to anatomical variations or physiological factors, but further investigation is needed to confirm this observation.

Our results agree with the ones in the literature, especially the ones derived from the cadaver studies.

### 4.2. Underwood’s Septa

Interest in the maxillary sinus septa has grown due to its association with the Schneider membrane perforation during surgery.

The last systematic reviews and meta-analyses, which gathered studies on CT scans and CBCTs, found a proportion of septa of 33.2% per sinus [47].

In the present study, our data disagree, since we observed their presence in 19% of the examined sample. These dense bone projections in the maxillary sinus can complicate surgical procedures and limit access to the sinus floor. However, septa may also have advantages, such as providing additional bone reinforcement for implant placement and facilitating faster bone formation. The septa’s height, location, and factor varied among individuals, but no significant differences were found between genders.

The septa orientation and position are crucial for the success of sinus floor lift procedures; indeed, Irinakis et al. [48] showed that the bucco–palatal orientation is the higher risk orientation for membrane perforation.

The findings of this study provide valuable insights into the clinical anatomy of the maxillary sinus, specifically regarding the vascular supply and the presence of septa: these anatomical details have significant implications for surgical procedures. They can help clinicians anticipate and avoid potential complications. CBCT imaging allows clinicians to assess the AAa and plan surgical interventions accordingly accurately. Additionally, understanding the presence and characteristics of Underwood’s septa can aid in optimizing surgical approaches and achieving successful outcomes.

It is worth mentioning that this study has limitations: the sample size was relatively small, and this study focused on a specific patient population. Future studies with larger sample sizes and diverse populations must validate these findings. In addition, the software used, available in clinical practices, did not allow a 3D approach, only a 2D analysis. Studies using more advanced technology allow the segmentation of the structures and morphometric analysis. Furthermore, a comprehensive analysis of other anatomical variations and their clinical implications in the maxillary sinus, such as the maxillary sinus hypoplasia, maxillary volume variations [49], and inferior meatus pneumatization [50] could provide further insights into this complex anatomical region [49,50].

## 5. Conclusions

Within its limitations, this study investigated the arterial blood supply and the presence of Underwood’s septa in the maxillary sinus using CBCT imaging. The findings contribute to the existing knowledge of the clinical anatomy of the maxillary sinus, providing clinicians with important information for surgical planning and avoiding potential complications. Further research is warranted to expand our understanding of the anatomical variations in this region and their impact on surgical outcomes.

## Figures and Tables

**Table 1:** Alveolar antral artery: incidence of the intra-osseous variant and comparison of length on cranio-caudal direction and medium-size branches of AAa between right side and left side.

AAa Intra-Osseous Variant (%)	AAa on the Left Sinus	AAa on the Right Sinus	*p*-Value (Chi-Square)
100	96	97	>0.05
**Parameters of AAa**	**Mean ± S. D. (mm)**		***p*** **-Value (Student *t*-Test)**
**AAa Length-right side**	24.06 ± 5.66		0.06 (1.85)
**AAa Length-left side**	23.21 ± 5.43		
**Cranio-caudal distance AAa-right side**	12.25 ± 3.84		0.007 (−2.72)
**Cranio-caudal distance AAa-left side**	13.44 ± 3.91		
**AAa Medium-size branches-right side**	1.07 ± 0.38		0.72 (0.36)
**AAa Medium-size branches-left side**	1.07 ± 0.33		

**Table 2:** Frequency of alveolar antral artery based on different medium-size branches, in the first superior molar area.

Medium-Size Branch	Number of Cases on Right Side	Number of Cases on Left Side	Frequency (%)
**≤1 mm**	52	44	48%
**range 1–2 mm**	47	56	51%
**>2 mm**	1	0	1%

**Table 3:** Student’s *t*-test of length on cranio-caudal direction of AAa between right side and left side and males and females, and of length of AAa between right side and left side and males and females.

Gender	Cranio-Caudal Distance of the Right AAa (Mean ± S.D.)	Cranio-Caudal Distance of the Left AAa (Mean ± S.D.)	*p* Value (Student’s *t*-Test)
M	11.66 ± 33.83	13.42 ± 43.31	>0.05
F	12.671 ± 41.23	13.46 ± 35.99	
**Gender**	**Length of the Right AAa (Mean ± S.D.)**	**Length of the Left AAa (Mean ± S.D.)**	***p* Value (Student’s *t*-Test)**
M	24.225 ± 48.54	24.141 ± 51.82	>0.05
F	23.947 ± 62.09	22.494 ± 55.61	

**Table 4:** Comparison of the caliber of the AAa. The caliber in the right hemiarch is statistically significant, being greater in male subjects.

Gender	Caliber of the Right AAa (Mean ± S.D.) (mm)	Caliber of the Left AAa (Mean ± S.D.) (mm)	*p* Value (Student’s *t*-Test)
M	1.18 ± 0.44	1.13 ± 0.33	>0.05
F	1.07 ± 0.31	1.02 ± 0.32	
***p* value (Student’s *t*-test)**	<0.05		

**Table 5:** Underwood’s septa frequency in the considered sample.

	Patients with Septa (n)	Single Septa (n)	Double Septa (n)	*p*-Value (Chi-Square between Males and Females)
**Total of patients**	15	11	4	>0.05
**Female patients**	9	6	3	
**Male patients**	6	5	1	

**Table 6:** Underwood’s septa in the right and left side.

	Single Septa (n)	Double Septa (n)
**Right side**	7	4
**Male patients**	5	1
**Female patients**	2	3
**Left side**	4	0
**Male patients**	0	0
**Female patients**	4	0

**Table 7:** Distribution of single septa: anterior–central–posterior area; right and left sides.

	Anterior (n)	Central (n)	Posterior (n)
**Right side**	4	2	1
**Male patients**	4	0	1
**Female patients**	0	2	0
**Left side**	3	1	0
**Male patients**	0	0	0
**Female patients**	3	1	0

**Table 8:** Distribution of double septa: anterior–central–posterior area; right and left sides.

	Anterior	Central	Posterior
**Right side**	0	3	1
**Male patients**	0	1	0
**Female patients**	0	2	0
**Left side**	0	0	0

## Data Availability

Data will be available upon reasonable request to the corresponding author.
